# The Link between Fetal Programming, Inflammation, Muscular Strength, and Blood Pressure

**DOI:** 10.1155/2015/710613

**Published:** 2015-09-27

**Authors:** Jose Lopez-Lopez, Patricio Lopez-Jaramillo, Paul A. Camacho, Diego Gomez-Arbelaez, Daniel D. Cohen

**Affiliations:** ^1^Dirección de Investigaciones, Fundacion Oftalmologica de Santander (FOSCAL), Floridablanca, Colombia; ^2^Facultad Ciencias de la Salud, Universidad de Santander (UDES), Bucaramanga, Colombia; ^3^Facultad de Medicina, Universidad Autonoma de Bucaramanga (UNAB), Bucaramanga, Colombia; ^4^División de Endocrinología, Escuela de Medicina, Universidad de Santiago de Compostela, 15782 Santiago de Compostela, Spain

## Abstract

Hypertension affects one billion individuals worldwide and is considered the leading cause of cardiovascular death, stroke, and myocardial infarction. This increase in the burden of hypertension and cardiovascular diseases (CVD) is principally driven by lifestyle changes such as increased hypercaloric diets and reduced physical activity producing an increase of obesity, insulin resistance, and low-grade inflammation. Visceral adipocytes are the principal source of proinflammatory cytokines and systemic inflammation participates in several steps in the development of CVD. However, maternal and infant malnutrition also persists as a major public health issue in low- to middle-income regions such as Latin America (LA). We propose that the increased rates of cardiovascular and metabolic diseases in these countries could be the result of the discrepancy between a restricted nutritional environment during fetal development and early life, and a nutritionally abundant environment during adulthood. Maternal undernutrition, which may manifest in lower birth weight offspring, appears to accentuate the relative risk of chronic disease at lower levels of adiposity. Therefore, LA populations may be more vulnerable to the pathogenic consequences of obesity than individuals with similar lifestyles in high-income countries, which may be mediated by higher levels of proinflammatory markers and lower levels of muscle mass and strength observed in low birth weight individuals.

## 1. Introduction

The prevalence of arterial hypertension and cardiovascular diseases (CVD) has grown significantly in recent years, particularly in low- and middle-income regions such as in Latin America [1]. In 2008, more than 1,400 million adults were overweight and 500 million were obese [2]. Hypertension affects one billion individuals worldwide and is considered the leading cause of cardiovascular death, stroke, and myocardial infarction [3]. Approximately 75 percent of people with hypertension live in developing countries where health resources are limited and there is a low level of awareness and control of the disease [4]. It is estimated that two thirds of all strokes and half of coronary heart disease events are attributable to the inadequate control of high blood pressure [5].

This increase in the burden of hypertension and CVD is principally driven by lifestyle changes such as increased hypercaloric diets and reduced physical activity. These lifestyle factors, combined with a genetic predisposition among the population of developing countries, have led to rapid increases in obesity, insulin resistance, and low-grade inflammation and its complications such as diabetes mellitus type 2 (DM2) and CVD [6].

## 2. Abdominal Obesity, Inflammatory Response, and Cardiovascular Risk

In low- to middle-income countries (LMICs), the rapid changes consequent to the urbanization process have produced an environment which discourages physical activity and encourages the consumption of energy dense diets, promoting the development of obesity and in particular abdominal obesity—a proxy measure of visceral adipose fat. Visceral adipocytes not only behave as a fat reserve but also as an endocrine organ and are the principal source of proinflammatory cytokines such as TNF-*α* and IL-6 [7]. These cytokines stimulate the production of acute phase reactants (C reactive protein [CRP], fibrinogen, and sialic acid) that promote systemic low-grade inflammation. Systemic inflammation participates in several steps in the development of CVD, including endothelial dysfunction, formation of the atheroma plaque, acute thrombotic complications, and hypertension [8, 9]. In the Colombian population, our group showed for the first time that high levels of CRP were associated with elevated blood pressure, leading to the hypothesis that low-grade inflammation is an independent risk factor for essential hypertension [10]. This data was confirmed in a subanalysis of the Women's Health Study, showing that basal levels of CRP were significantly higher in women with hypertension than those that did not develop hypertension and that CRP levels were significantly associated with the risk of hypertension [11]. In addition, studies conducted in the Andean population have demonstrated that CRP and other markers of low degree inflammation are elevated in subjects with pregnancy-induced hypertension [12]. In this population, we observed that low degree inflammation is a critical factor in the development of myocardial infarction [8]. Also, the association between inflammation markers and metabolic syndrome has been observed in a similar population in China [13].

Moreover, in children we found a significant positive correlation between body mass index (BMI), blood pressure, and CRP [14]. Notably, CRP concentrations of the second tertile of BMI in Colombian children were as high as those reported in overweight and obese Caucasian-American and European children in a similar age range [15, 16].

On the basis of these results, we have proposed that LMIC populations are predisposed to produce an inflammatory response at lower body fat levels than Caucasian populations, a consequence of their shorter period of exposure to western lifestyles.

Moreover, we proposed that a shorter period of exposure leads to a delay in the process of adaptation to these lifestyle changes, and in turn to a greater risk of low-grade inflammation and insulin resistance at lower levels of abdominal obesity [17]. The process of adaptation is the result of epigenetic changes in the expression of the genes that regulate the production of angiotensin II (Ang II), leptin, and adiponectin in adipose tissue. It is well known that Ang II is a strong vasoconstrictor that regulates vascular tone, sodium, and water retention and increases blood pressure. In addition, it has been demonstrated that it blocks intracellular insulin signaling pathways, predisposing to hyperglycemia, and the development of DM2. Moreover, Ang II stimulates a state of oxidative stress and an increased inflammatory response [18]. Jandeleit-Dahm et al. [19] found that, in obese, insulin-resistant, or hypertensive subjects, treatment with Ang II AT1 receptor antagonists reduces insulin resistance and reduces new cases of DM2, suggesting a relationship between Ang II, leptin, and adiponectin. Moreover, the beneficial effects of AT1 receptor blockers on adipose tissue mass and insulin resistance in obesity could be related to the enhancement of adiponectin expression, the reduction of leptin expression, and the concomitant correction of the leptin/adiponectin imbalance [20]. It has been shown in transgenic mice that the overexpression of human adiponectin was associated with the suppression of fat accumulation and with protection against mortality associated high-calorie diet [21]. Interestingly, regional differences in the expression of adiponectin [22–26] have been shown in several studies, leading to the proposal that, in developed/high-income countries where westernized lifestyles have been present for many years with a prolonged period of adaptation, the overexpression of adiponectin has been promoted. However, there is a paucity of data, and more research is required to evaluate this hypothesis.

## 3. Fetal Programming and the Risk of Cardiovascular Diseases

The increase in the prevalence of overweight and obesity in Latin American adults, adolescents, and children is evident [27–29]. However, maternal and infant malnutrition persists as a major public health issue in this region [30]. The fetal programming concept suggests that a poor in-uterus nutritional environment influences the development of the organism in such a way as to prepare it to live in nutrient restricted conditions. However, the organism is metabolically less well prepared for exposure to a very different, “energy rich” environment during growth and development and in later life, and it is this mismatch between a poor early life and later obesogenic environment that is thought to lead to increased susceptibility to insulin resistance, low-grade inflammation, and chronic disease such as DM2 and CVD [31]. This is particularly so when this unfavorable environment interacts with a genetic predisposition. The body of literature from human epidemiological studies and dietary interventions in animal models has provided considerable evidence to suggest that maternal nutritional imbalance and metabolic disturbances during critical periods of intrauterine development, may have a persistent and intergenerational effect on the health of offspring and on the risk of diseases such as obesity, CVD, diabetes, hypertension, asthma, and cancer [6, 32, 33]. In early work, it was found that older men with low birth weight (LBW) had a sixfold greater risk of DM2 than those with normal birth weight. Evidence to suggest that the risk of developing a number of these diseases in adulthood is influenced by both the duration and the timing of nutritional deficit during pregnancy also comes from studies such as the Dutch Winter Hunger cohort. This study showed that the offspring of mothers who experienced undernutrition during the early stage of pregnancy were at greater risk for the development of CVD than those who had the nutritional deficit at a more advanced stage of pregnancy [34, 35]. Not only fetal but also neonatal periods have been identified as critical for the development and growth of the systems involved in cardiometabolic pathways; for example, a negative effect on the number and secretor function of pancreatic beta-cells has been observed in rats [36, 37]. Adults who had both LBW and higher rates of growth at 7 years of age had a further increased risk of developing DM2 [38], showing that maternal undernutrition also interacts with catch-up growth to determine disease risk.

Franco et al. [39] demonstrated that circulating noradrenaline levels were significantly higher in children with LBW for gestational age compared with normal birth weight children and noted that there was a significant association between circulating levels of AII, angiotensin-converting enzyme (ACE), and blood pressure levels. In a Colombian pediatric population, we found a positive correlation between BMI, systolic blood pressure (SBP), and CRP, further reinforcing the idea of a link between obesity, elevated inflammatory markers, endothelial dysfunction, and elevated blood pressure [14]. Based on this data, we proposed that the increased rates of cardiovascular and metabolic diseases currently observed in LA could be the result of the discrepancy between the restricted nutritional environment during fetal development and early life and the nutritionally abundant environment individuals are exposed to in adulthood.

## 4. Low Birth Weight, Muscular Strength, and Inflammation

The Pan-European HELENA study showed that, in youth, poor performance in tests of both cardiorespiratory fitness (CRF) and muscular fitness is associated with elevated metabolic and cardiovascular risk factors [40]. This and other studies have shown that overweight and obese adolescents have a better metabolic profile if they have adequate muscular or cardiorespiratory fitness [41].

Critically, in terms of primary prevention, in a prospective study of 1 million adolescents, low handgrip strength was identified as a predictor of premature mortality in adulthood, an association of similar magnitude as the well-established risk factors BMI and blood pressure [42]. There is now substantial evidence, mainly from high-income countries, that low muscle strength is a risk factor for chronic disease and a predictor of CVD and all-cause mortality both in initially healthy adults [43] and in those with existing disease, including hypertension [44]. Furthermore, we recently showed in an international cohort DM2 and pre-DM2 patients [45], which included people from LA and other LMIC regions, that lower handgrip strength (HG), a widely used index of whole body muscle strength, was a powerful predictor of cardiovascular and all-cause mortality over 6 years of follow-up.

**Figure 1:**
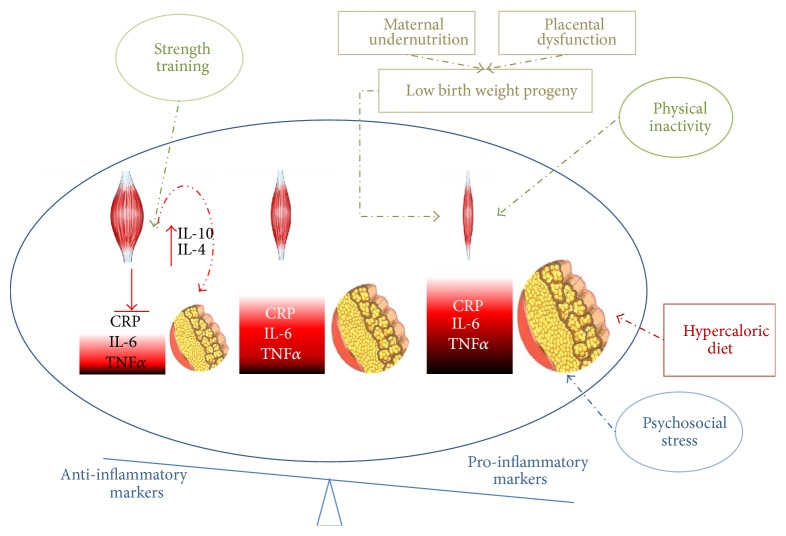
The opposing influences of muscle mass and fat mass on the pro-inflammatory : anti-inflammatory balance. The middle condition shows normal muscle mass and fat mass. The condition on the right shows the impact of low birth weight and lower muscle mass/strength interacting with lifestyle influences such as inactivity, which also promotes lower muscle mass/strength and hypercaloric diets, promoting increased fat mass. Larger fat mass stimulates pro-inflammatory adipokine release and reduces anti-inflammatory adipokine release, while lower muscle mass/strength and lower physical activity are associated with higher pro-inflammatory adipokine and lower anti-inflammatory myokine release, respectively. To the left is the condition whereby the influence of strength training stimulates muscle mass/strength, associated with a lower inflammatory burden and which also promotes the secretion of anti-inflammatory myokines such as IL-4 and IL-10.

While the precise mechanism by which muscle strength and or mass are protective against cardiometabolic diseases is not clear, it is suggested that this may be mediated by the inverse associations between strength and classic risk factors such as HOMA index, triglycerides, and blood pressure, positive associations with markers of vascular function [46], and lower arterial stiffness [47] and/or its inverse associations with inflammatory markers such as CRP and TNF-*α* [48]. Indeed, in children as young as 9, Steene-Johannessen et al. [49] reported that independent of adiposity and CRF, higher muscular strength was associated with lower levels of markers of chronic inflammation such as CRP, leptin, and TNF-*α*. In the HELENA study, Artero et al. [50] found an inverse association between muscular strength and CRP adolescents after adjusting for gender, age, cardiorespiratory fitness, maturation, and socioeconomic status. There is little data regarding these interactions from children or adults in low- to middle-income countries. However, we recently demonstrated in the ACFIES study of fitness and cardiometabolic risk factors in Colombian schoolchildren, that handgrip strength was inversely associated CRP, blood pressure, HOMA index, and a composite metabolic risk score [51]. We found that the inverse associations between handgrip strength and CRP as well as other markers of cardiometabolic risk were significant among the children in the highest tertile of % fat but not among those in the lower and middle tertiles. Artero et al. [44, 50] and Steene-Johannessen et al. [41] also found stronger inverse relationships between muscle strength and metabolic risk factors in overweight/obese children. Thus, it appears that adequate muscle strength/mass is of increased importance in individuals with higher fat mass, particularly those with higher visceral fat mass [52], presumably due to the burden of chronic inflammation associated with this adipose depot. Higher visceral fat is associated both with higher levels of pro-inflammatory adipokines such as CRP and TNF-*α* and with lower anti-inflammatory adipokines such as adiponectin. In contrast, muscle strength and mass are associated with lower CRP and TNF-*α*. Moreover, muscle tissue produces and secretes a variety of anti-inflammatory cytokines (myokines) which are released during exercise and oppose the effects of the pro-inflammatory adipokines [53, 54] on glucose and fat metabolism [55]. Furthermore, there is evidence that strength training may be particularly effective in promoting beneficial changes in the pro-inflammatory: anti-inflammatory balance [55, 56]. In DM2 patients, for example, 20 mins of strength training and 40 mins of aerobic training led to larger decreases in CRP than 60 mins of exclusively aerobic training [55]. Furthermore, increases in the anti-inflammatory IL-4 and IL-10 were only observed in the group that also participated in strength training [55]. Notably, the inverse relationship between muscle and chronic inflammation appears to be bidirectional, as higher levels of visceral fat and chronic inflammatory markers such as CRP are predictors of future decline in muscle mass and strength [57]. This could be of particular relevance to LMIC populations who may have lower muscle strength, a higher inflammatory burden and susceptibility to the pro-inflammatory effects of obesity and associated metabolic dysfunction [14]. Hispanic, African, and Asian origin is independently associated with higher CRP levels [58] and low birth weight, which is more common in LMIC's, is also associated with higher levels of CRP [14] (Figure 1).

The deficit in body size or “somatic capital” in the low birth weight neonate is almost entirely accounted for by lower fat-free mass [59] and a positive association between birth weight and fat-free mass is a highly consistent finding [60] observed across the lifecycle. Muscle fiber number and type are set in-utero and experimentally induced fetal undernutrition in animals is associated with lower muscle fiber number [61]. In humans, LBW is also associated with alterations in muscle morphology [62] and deficits in muscle signaling proteins [63], alterations that negatively affect muscle glucose and fat metabolism and may precede metabolic abnormalities and increase susceptibility to obesity and comorbidities. Interestingly, one of the alterations identified in LBW young adults, is a defect in the activation of Akt-1 [64], a signaling protein involved both in muscle protein synthesis and in insulin signaling [65, 66]. This finding may help explain the persistent effects of fetal undernutrition on attenuated muscle mass accretion via impaired anabolic signaling in muscle [67].

Functional indicators of fat-free mass, particularly muscle strength, are also consistently observed in LBW [68–70], and deficits in other aspects of muscle performance, such as muscle endurance [71], speed, and motor performance [71] are also reported. Some reports also show lower cardiorespiratory fitness (CRF) in LBW individuals [72], but this deficit appears to be accounted for by lower muscle mass [73] and Brøns et al. [73] observed that LBW was not associated with impaired mitochondrial function. Therefore, it appears that in LBW, neuromuscular performance and morphological characteristics of muscle, size and fiber type, may be relatively more affected than oxidative capacity. Two specific findings of the ACFIES study [51] also appear to suggest an increased emphasis on ensuring adequate strength and muscle mass in LBW individuals within transitional populations such as LA, as a means of counteracting its negative cardiometabolic health consequences. Firstly, in Colombian children relative to the associations between CRF and cardiometabolic risk factors, associations between HG and these risk factors were stronger, contrary to what has generally been observed in previous studies in European children, examples of comparable European data are shown in Table 1. Furthermore, adjusting for HG substantially reduced the association between CRF and cardiometabolic risk factors (from *r* = −0.2189, *p* < 0.001, to *r* = −0.115, *p* < 0.05), while adjusting for CRF had little effect on the association between HG and these risk factors (*r* = −0.357, *p* < 0.001 to *r* = −0.298  *p* < 0.05). Secondly, we noted that in the subsample of children for whom we could obtain reliable birth weight records, there was stronger inverse association between HG and metabolic risk score: *r* = −0.599, *p* = 0.002 compared to *r* = −0.383, *p* < 0.001, in the normal birth weight schoolchildren.

Based on these findings and other observations, we therefore suggest that muscle strength/mass may have elevated importance to cardiometabolic health in our population. However, our findings, based on a sample from a single urban public school, might not be generalizable, and interactions between birth weight, muscular strength and cardiometabolic health should be examined in larger samples of Colombian or other Latin American youth.

While there is now substantial evidence that as early as childhood, low muscle mass/strength is associated with elevated traditional cardiovascular risk factors and a more pro-inflammatory cytokine profile. However, given the associations between low birth weight and both a poor cardiometabolic profile and later chronic disease [31, 32, 34, 35, 38], it is possible that low muscle mass/strength is simply a marker of risk related to poor early life conditions and one component of the low birth weight phenotype rather than an active component of cardiovascular risk [74].

## 5. Conclusion

Obesity is recognized as an international epidemic driving rapid increases in chronic disease such as DM2 and hypertension, and putting an increasing burden on the less well-resourced health systems of LMIC's undergoing nutrition transition. In particular, Low-grade chronic inflammation promoted by visceral adiposity is one of the principle mediators of pathogenic changes in the endothelial/vascular system which ultimately lead to hypertension. Despite the increasing adoption of westernized, obesogenic lifestyles within LMICs, characterized by increased consumption of high energy density diets and reduced physical activity, these populations are also still burdened by maternal undernutrition. Furthermore, early life insults, which may manifest in lower birth weight offspring, appear to accentuate the relative risk of chronic disease at lower levels of adiposity. Thus, LMIC populations may be more vulnerable to the pathogenic consequences of obesity than individuals with similar lifestyles in high-income countries. While epidemiological studies show a strong association between low birth weight and later chronic disease, evidence that LBW individuals also show higher level of proinflammatory markers and lower levels of muscle mass and strength suggests potential contributory pathways. Moreover, muscle and visceral fat tissue have opposing effects on metabolism, mediated by their promotion of pro- and anti-inflammatory milieu, respectively. Physical activity is universally recommended for the prevention of chronic disease and should include both aerobic type and strengthening activity. In LMIC populations with a higher prevalence of specific cardiometabolic risk factors linked to low birth weight, specifically; inflammation and lower muscle mass/strength, we suggest that ensuring adequate muscle mass and muscle strength may be of particular importance from a public health and chronic disease prevention perspective.

## Figures and Tables

**Table 1:** Relationship between handgrip strength (HG), cardiorespiratory fitness (CRF) and cardiometabolic risk factors in children and adolescents.

	ACFIES (Colombian)		HELENA (Pan-European)		PANCS (Norwegian)	
	HG	CRF	HG	CRF	HG	CRF
SBP	−0.077	0.003	−0.090^*∗*^	−0.046	−0.06^*∗*^	−0.14^*∗∗*^
DBP	−0.196^*∗∗∗*^	−0.148^*∗∗∗*^	—	—	—	—
HOMA	−0.178^*∗∗∗*^	−0.162^*∗∗∗*^	−0.186^*∗∗∗*^	−0.265^*∗∗∗*^	−0.19^*∗∗∗*^	−0.31^*∗∗∗*^
TG	−0.104^*∗*^	−0.037	−0.119^*∗∗*^	−0.168^*∗∗∗*^	−0.07^*∗∗*^	−0.20^*∗∗∗*^
CRP	−0.107^*∗*^	−0.045	−0.139^*∗∗∗*^	−0.141^*∗∗∗*^		

Values shown are *β* coefficients or *r* values for the relationship between HG (/kg bodyweight) or CRF and markers of cardiometabolic risk. HG:Handgrip/kg body weight; CRF: ACFIES, HELENA estimated CRF using a 20 m shuttle run test, PANCS directly assessed CRF using a progressive cycle ergometer test.

SBP: systolic blood pressure; DBP: diastolic blood pressure; HOMA: Homeostasis Model assessment index; TG: triglyceride; CRP: C reactive protein.

ACFIES data is Cohen et al. 2014 [51], HELENA is Artero et al. 2011 [40] except for CRP, which is Artero et al. 2014 [40], and PANCS is Steene-Johannessen et al. 2009 [41].

^*∗*^ *p* < 0.05, ^*∗∗*^ *p* < 0.01, and ^*∗∗∗*^ *p* < 0.001.
